# Adjunctive povidone-iodine for *Acanthamoeba* keratitis: a randomized trial

**DOI:** 10.1128/aac.01512-25

**Published:** 2026-03-31

**Authors:** Revathi Rajaraman, Isabell Kassaye, Sankalp S. Sharma, N. Venkatesh Prajna, Prajna Lalitha, Arunkumar Panigrahi, Ram Rammohan, Krisianne M. Aromin, Catherine Cook, Travis C. Porco, Thomas M. Lietman, Gerami D. Seitzman, Jeremy D. Keenan

**Affiliations:** 1 Cornea Department, Aravind Eye Hospital 75196 , Coimbatore, India; 2 Francis I Proctor Foundation, University of California 8785, San Francisco, California, USA; 3 Department of Cornea and Refractive Surgery, Aravind Eye Hospital 29954, Madurai, India; 4 Department of Microbiology, Aravind Eye Hospital 75196 , Madurai, India; 5 Department of Microbiology, Aravind Eye Hospital 29954, Coimbatore, India; 6 Department of Ophthalmology, University of California 8785, San Francisco, California, USA; The Children's Hospital of Philadelphia, Philadelphia, Pennsylvania, USA

**Keywords:** povidone-iodine, *Acanthamoeba* keratitis, microbiological techniques

## Abstract

The aim of this study was to determine the antiamoebic efficacy of povidone-iodine when used as an adjunct to topical biguanide therapy for *Acanthamoeba* keratitis. Patients with smear- or culture-positive *Acanthamoeba* keratitis received topical chlorhexidine 0.04%, half-hourly for 2 days, then hourly for days 3–6, then eight times per day for days 7–28. Participants were randomized in a 1:1 ratio to receive either adjunctive topical povidone-iodine at the same frequency as chlorhexidine or no additional therapy (i.e., control group). Povidone-iodine was initially administered as a 2.5% solution but was reduced to a 1% solution after several reports of intolerance. The primary outcome was *Acanthamoeba* growth from the culture of corneal scrapings at 1, 2, and 4 weeks after randomization, as assessed by laboratory staff masked to treatment allocation. Of 49 patients enrolled, 25 were randomized to adjunctive povidone-iodine (mean age 48 [SD 16] years; 32% female) and 24 to control (mean age 43 [15] years; 33% female). Four (16%) participants discontinued 2.5% povidone-iodine prematurely due to intolerance. The proportion of participants with a positive culture during the initial 4 weeks of antiamoebic treatment was not significantly different in the povidone-iodine group (6/18 [33%] at 4 weeks) and control group (5/16 [31%] at 4 weeks) (RR 0.90, 95%CI: 0.35–2.29; *P* = 0.82). When used as an adjunctive to standard biguanide therapy, povidone-iodine did not markedly accelerate the clearance of *Acanthamoeba* organisms from the corneal surface over the initial month of therapy.

This study is registered with ClinicalTrials.gov as NCT03484507.

## INTRODUCTION

*Acanthamoeba* keratitis (AK) is a rare corneal infection with few treatment options (1). *Acanthamoeba* exists in two forms: a metabolically active motile trophozoite and a dormant cyst (2). While trophozoites are relatively susceptible to a variety of topical antimicrobial agents, cysts, with their double-walled structure, are more resistant to physical and chemical damage, making them more difficult to eradicate from the cornea. The current mainstay of therapy is a biguanide agent such as chlorhexidine or polyhexamethylene biguanide (PHMB), with the latter agent recently approved in Europe at a concentration of 0.08% for the treatment of AK (3, 4). Many cornea specialists add a variety of adjunctive topical treatments, most commonly diamidines (e.g., propamidine and hexamidine) and azoles, although the effectiveness of adjunctive therapies has not been well-characterized (5). None of these medications were developed specifically for AK, but they have each demonstrated favorable antiacanthamoebic susceptibility profiles *in vitro* (6). Several laboratory studies have found povidone-iodine to be effective for killing *Acanthamoeba* cysts *in vitro* (7–12). Other studies have failed to find an effect of povidone-iodine on *Acanthamoeba* (13, 14). Our previous laboratory studies found povidone-iodine to be an extremely effective cysticidal agent, making it a promising treatment option, given its wide availability and low cost. However, its role in AK therapy is unclear, given the mixed results of other laboratory studies as well as its poor corneal penetration (15).

The objective of this study was to evaluate the antiamoebic efficacy of 2.5% povidone-iodine when used as an adjunctive therapy for AK. We chose this concentration of povidone-iodine based on the rationale that a relatively high concentration might penetrate more deeply into the cornea and given prior studies that found this dose to be safe (16). We hypothesized that participants treated with a combination of chlorhexidine and povidone-iodine would have more rapid microbial clearance of *Acanthamoeba* organisms from the corneal surface compared with patients who were treated with chlorhexidine alone.

## MATERIALS AND METHODS

### Trial design

This study was a parallel group randomized clinical trial conducted from 13 January 2018 to 22 September 2022, in which participants with AK were randomized to receive either chlorhexidine 0.04% monotherapy or chlorhexidine 0.04% plus adjunctive povidone-iodine 2.5% (ClinicalTrials.gov NCT03484507). The primary outcome was microbiological evidence of *Acanthamoeba* on culture from corneal scrapings collected at weeks 1, 2, and 4. The trial protocol and statistical analysis plan are available at https://osf.io/5rsmp; the only major change to the protocol after starting the trial was that the concentration of povidone-iodine was reduced from 2.5% to 1% due to frequent intolerance of the higher dose.

### Study participants

Participants were enrolled at the Madurai and Coimbatore tertiary eye hospitals of the Aravind Eye Care System in Tamil Nadu, India. Patients aged 13 years or older with corneal scrapings positive for *Acanthamoeba* by culture or smear were eligible for enrollment. Exclusion criteria were interstitial or viral keratitis on history or examination, corneal perforation, therapeutic keratoplasty for AK, and/or unwillingness or inability to follow-up.

### Interventions

All participants received topical 0.04% chlorhexidine, one drop half-hourly while awake for the first 2 days, then one drop hourly while awake for days 3–6, then one drop eight times per day for days 7–28. The 0.04% concentration and dosing frequency were based on the clinical experience of the investigators and others (1). Although the trial endpoint was day 28, chlorhexidine was continued at the discretion of the treating doctor until the ulcer had healed, potentially many months longer. Participants randomized to povidone-iodine were instructed to take the same frequency of povidone-iodine as chlorhexidine, starting immediately after randomization. Note that participants without a prior diagnosis of AK would have started chlorhexidine and povidone-iodine the same day, whereas others may have been treated with antiamoebic therapy for some duration of time prior to starting povidone-iodine. A 2.5% solution of povidone-iodine was initially given based on numerous prior reports that found this concentration to be safe, but after 39 total participant enrollments, this was reduced to 1% due to occasional intolerance of the higher dose (16). Participants in both arms who were taking more than one type of eye drop were instructed to wait at least 15 min before instilling the next medication. No placebo was attempted for the control group since povidone-iodine has a dark color that was difficult to match. Both chlorhexidine and povidone-iodine were formulated by the Microbiology Laboratory at Aravind Eye Hospital-Madurai, using stock solutions of chlorhexidine diguanide solution 20% (Sigma-Aldrich, St. Louis, USA) and povidone-iodine 5% (Aurolab, Tamil Nadu, India), respectively. The resulting concentrations were not confirmed by external testing, but stock solutions of the commercially available products were subject to quality controls, and the laboratory staff performing the dilutions were experienced in this routine task.

### Outcomes

The pre-specified primary outcome was the clearance of *Acanthamoeba* identified on culture of corneal scrapings at 1, 2, and 4 weeks after randomization. Corneal scrapings were plated on non-nutrient agar overlaid with *Escherichia coli*, incubated at 37°C, and observed with an inverted microscope daily for 7 days to detect the growth of excysted trophozoites. Each study site had an experienced microbiology staff who routinely performed *Acanthamoeba* cultures for clinical care. Participants had corneal scrapings performed at study visits at week 1, week 2, and week 4 whether or not they had a corneal epithelial defect at the time and regardless of the results of any prior scrapings. Although it was expected that most participants would not have healed by the 1-month endpoint of the trial, several secondary clinical outcomes were pre-specified, including best spectacle corrected visual acuity (BSCVA) at 4 weeks, absence of ocular inflammation or epithelial defect at 4 weeks, and time until re-epithelialization (defined as the first visit without an epithelial defect, censored at 4 weeks). Microbial clearance was selected as the primary outcome since a main goal of an antimicrobial agent is to clear the infectious organism as quickly as possible. This outcome was thought to be more likely to result in a large treatment effect, given preliminary *in vitro* studies—an important consideration given the impracticality of enrolling a large sample size for this uncommon infection (9). Harms were assessed systematically at each follow-up visit.

### Sample size

Assumptions for power calculations were based on a retrospective study of *Acanthamoeba* clearance in biguanide-treated AK, which estimated a median clearance time of approximately 6 weeks, as well as on unpublished preliminary data collected at Aravind Eye Hospital that estimated a median clearance time of approximately 4 weeks (17). Assuming one-half of participants treated with chlorhexidine monotherapy would clear *Acanthamoeba* by 4 weeks, enrolling 22 participants per treatment arm would provide approximately 80% power to detect a 40% difference in culture positivity at 4 weeks, given an alpha of 0.05 and 5% loss to follow-up over the 4-week period. While relatively large, this effect size was thought plausible given *in vitro* studies that found povidone-iodine to be considerably more cysticidal than the biguanide PHMB (7, 9).

### Randomization and masking

Randomization was completed using the sample function in the statistical package R (R Foundation for Statistical Computing, Vienna, Austria), using default settings for pseudorandom number generation. Each participant was assigned a unique trial ID. Participants were randomized in a 1:1 ratio to the two treatment groups, with block randomization in randomly permuted blocks of two, four, and six at each enrollment site. The trial biostatistician created the allocation sequences, and a study coordinator not involved with trial implementation uploaded the sequences to the REDCap randomization module to ensure concealed allocation. The study personnel responsible for the assessment of the primary outcome (i.e., the microbiology laboratory staff) were masked to the randomization allocation by labeling biological materials with a study identifier but without patient-identifying information. Study personnel collecting BSCVA data were masked. The study coordinator, doctors, and participants were not masked.

### Statistical methods

The presence of *Acanthamoeba* on culture at all time points was modeled in a repeated measures analysis with mixed effects robust Poisson regression with terms for treatment arm, time, and the treatment by time interaction, and a random intercept for participant. Similar methods were used for several non-prespecified subgroup analyses. BSCVA was modeled in a linear regression adjusted for presenting Logarithm of the Minimum Angle of Resolution (logMAR) visual acuity at enrollment. Time to re-epithelialization was modeled in a Cox proportional hazards model. Statistical significance for the primary outcome was assessed by Monte Carlo permutation (10,000 replications) at a significance level of 0.05; secondary outcomes were considered hypothesis-generating and also assessed at a significance level of 0.05. Complete-case superiority analyses were conducted following intention-to-treat principles; the primary mixed effects model allowed inclusion of all non-missing observations, regardless of whether missing data were present at any of the other study visits. No interim analysis was performed. Data management was done in R version 4; statistical analyses were done in Stata 18. Data are available at https://osf.io/qhbu7.

## RESULTS

**Fig 1:**
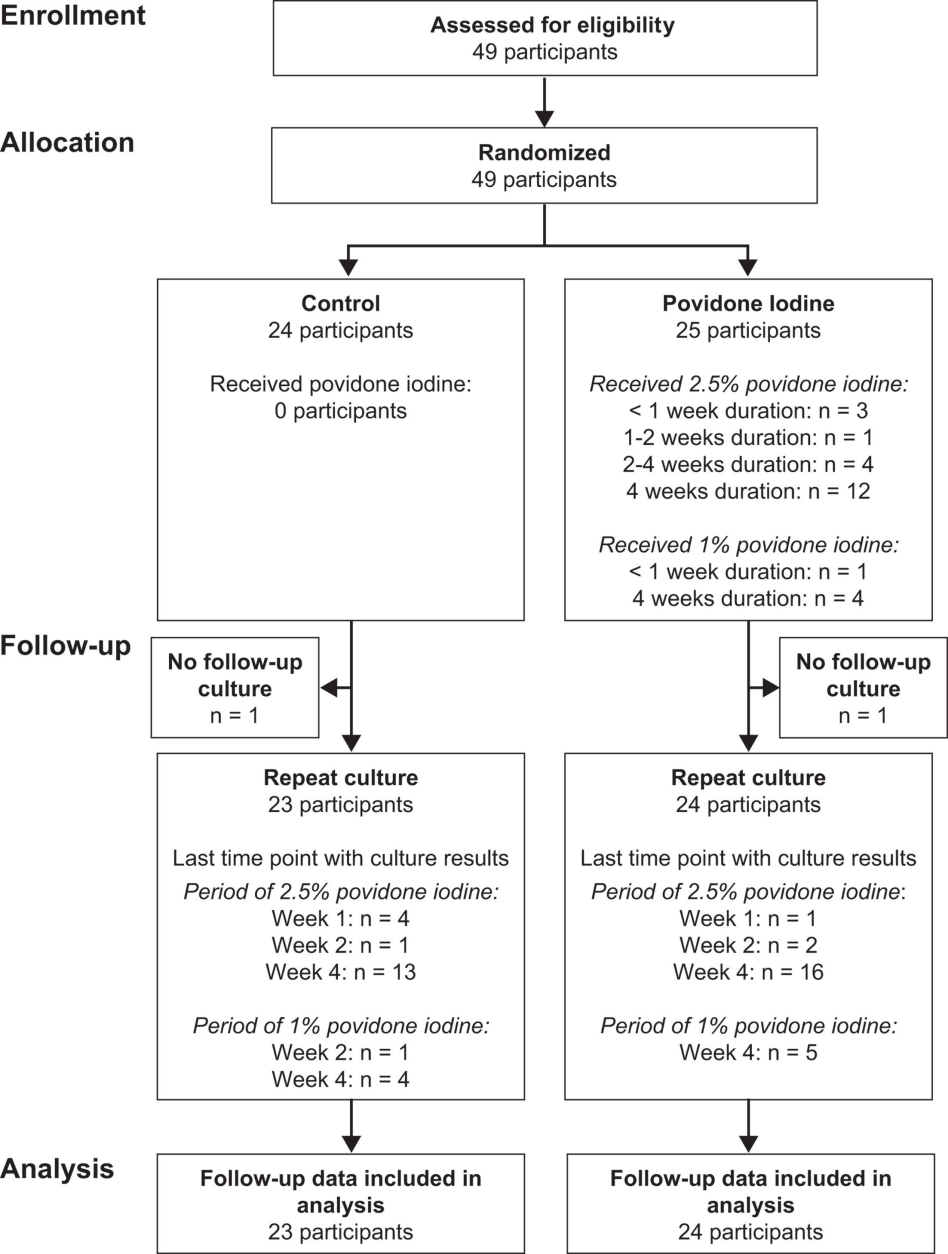
Flow diagram. Participants with *Acanthamoeba* on smear or culture were randomized to adjunctive povidone-iodine or a control arm that did not receive povidone-iodine. The povidone-iodine group initially received 2.5% povidone-iodine, but after reports of intolerance, this was changed to 1% povidone-iodine partway through the trial. The primary outcome was repeat culture from corneal scrapings, performed at weeks 1, 2, and 4; the diagram shows the last visit at which culture results were available.

In total, 49 eyes from 49 participants were enrolled, of which 30 were positive on culture and smear, 8 were positive on culture alone, and 11 were positive on smear alone. A total of 25 eyes were randomized to the adjunctive povidone-iodine group and 24 eyes to the control group (Fig. 1). Baseline characteristics were balanced in the two treatment arms (Table 1). All participants received their assigned allocation. Self-reported treatment adherence to chlorhexidine was balanced between the two arms, and adherence to povidone-iodine was acceptable, with a mean of 11.1 (SD 7.9) drops per day at week 1, 9.3 (SD 6.3) drops per day at week 2, and 7.1 (SD 6.1) drops per day at week 4 (Table S1). An additional antiamoebic agent was started at some point over the study period in two (8%) participants in the control arm and three (12%) participants in the povidone-iodine arm; the additional agent was PHMB in all cases.

**TABLE 1:** Baseline characteristics^*a*^

	Treatment arm	
Baseline characteristic	Control (*N* = 24)	Povidone-iodine (*N* = 25)
Demographics		
Female, no. (%)	8 (33%)	8 (32%)
Age in years, mean (SD)	43 (15)	48 (16)
Agricultural worker, no. (%)	10 (42%)	12 (48%)
Medical history		
Contact lens wearer, no. (%)	2 (8%)	3 (12%)
Days since symptoms started, mean (SD)	30 (21)	28 (25)
Recent ocular trauma no. (%)	7 (24%)	8 (32%)
Concurrent medications, no. (%)		
Topical antiamoebic	10 (42%)	6 (24%)
Topical steroid	2 (8%)	4 (16%)
Topical antifungal	8 (33%)	12 (48%)
Topical antiviral	2 (8%)	1 (4%)
Clinical exam		
Right eye, no. (%)	14 (58%)	15 (60%)
logMAR visual acuity, mean (SD)	1.4 (0.5)	1.3 (0.5)
Reduced corneal sensation, no. (%)	12 (50%)	15 (60%)
Pseudodendrites, no. (%)	4 (17%)	1 (4%)
Epitheliopathy without stromal disease, no. (%)	4 (17%)	5 (20%)
Epithelial defect size in mm, mean (SD)	4.7 (1.9)	5.4 (2.8)
Ring infiltrate, no. (%)	16 (67%)	20 (80%)
Scleritis, no. (%)	1 (4%)	2 (8%)
Microbiology, no. (%)		
* Acanthamoeba* smear-positive, culture-negative	7 (29%)	4 (16%)
* Acanthamoeba* smear-negative, culture-positive	4 (17%)	4 (16%)
* Acanthamoeba* smear-positive, culture-positive	13 (54%)	17 (68%)
Fungus culture-positive	2 (8%)	0 (0%)
Bacteria culture-positive	0 (0%)	0 (0%)

^*a*^ logMAR, Logarithm of the Minimum Angle of Resolution.

**Fig 2:**
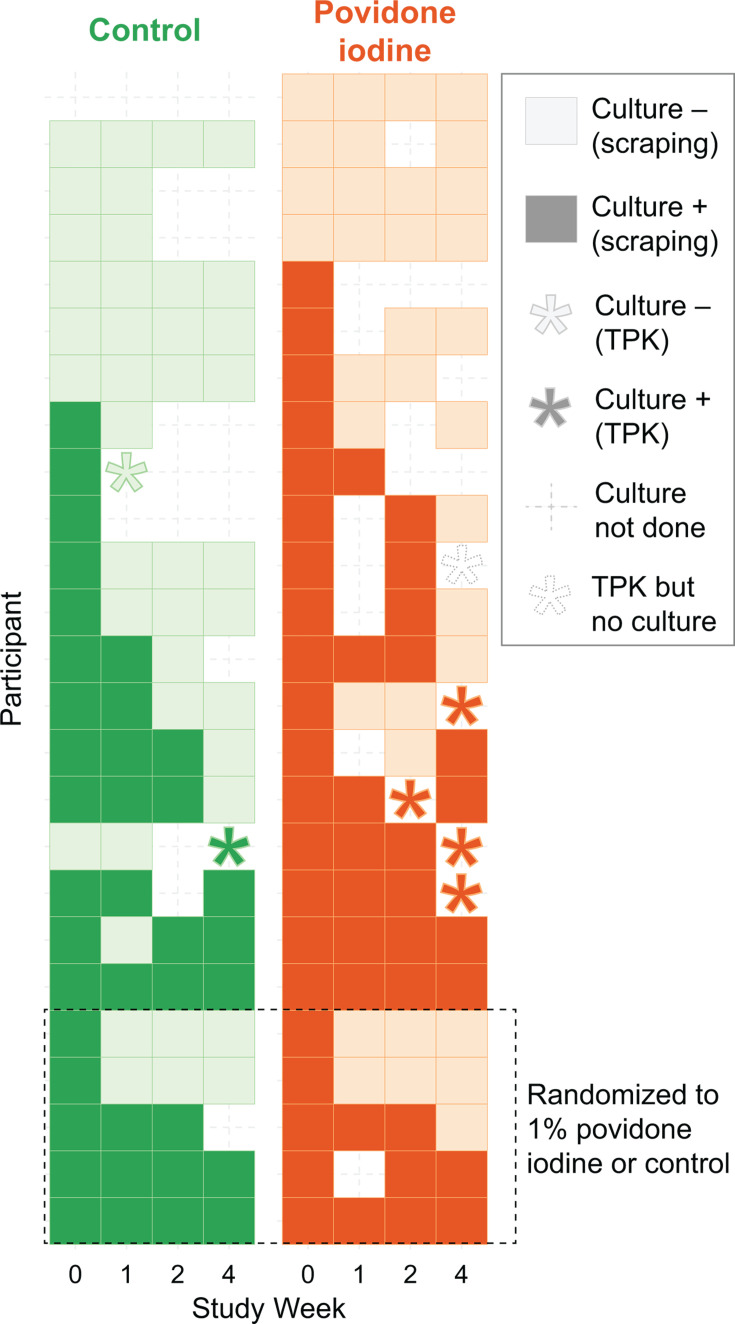
Participant-specific culture results across the four study visits. Darkly shaded tiles indicate a positive result on *Acanthamoeba* culture taken from corneal scrapings. Lightly shaded tiles indicate a negative result from corneal scrapings. Missing tiles indicate that no culture was performed. Asterisks represent therapeutic keratoplasty events, with darkly shaded symbols indicating a positive culture result, lightly shaded symbols indicating a negative culture result, and dashed symbols indicating missing culture data. Initially, participants were randomized to 2.5% povidone-iodine or no adjunctive treatment, but due to occasional drug intolerance, the final 10 participants were randomized to 1% povidone-iodine or no adjunctive treatment.

Repeat corneal scrapings for culture were performed in 40 participants at week 1, 37 participants at week 2, and 34 participants at week 4, with missing data relatively balanced between the two treatment arms (Fig. 2). Positive *Acanthamoeba* cultures were observed in 9 of 18 (50%) participants in the povidone-iodine group and 9 of 22 (41%) participants in the control group at week 1, 11 of 20 (55%) participants in the povidone-iodine group and 7 of 17 (41%) participants in the control group at week 2, and 6 of 18 (33%) participants in the povidone-iodine group and 5 of 16 (31%) participants in the control group at week 4 (Fig. S1). The difference between the two treatment groups was not statistically significant when all study visits were modeled (permutation *P* = 0.82, primary analysis), with an estimated incidence rate ratio (IRR) at day 28 of 0.90 (95%CI: 0.35–2.29) for the povidone-iodine group relative to control.

Of 21 eyes in the povidone-iodine group and 23 eyes in the control group with an epithelial defect at baseline, 4 and 5 had re-epithelialized by week 4, respectively (hazard ratio 0.94, 95%CI: 0.25–3.49; *P* = 0.92). Clinical resolution, defined as complete re-epithelialization and absence of ocular inflammation, was observed by week 4 in none of the eyes in the povidone-iodine group and one eye in the control group (*P* = 0.48, Fisher’s exact test). BSCVA was available for 21 participants in each group at the 4-week visit, with a mean logMAR of 1.42 (approximate Snellen equivalent 20/500; 95%CI: 1.12–1.73) in the povidone-iodine group and 1.49 (approximate Snellen equivalent 20/600; 95%CI: 1.28–1.70) in the control group. When adjusted for baseline vision, 4-week BSCVA was 0.9 lines worse (95%CI −1.7 lines better to 3.5 lines worse) in the povidone-iodine group compared with the control group at 4 weeks (*P* = 0.51).

Therapeutic penetrating keratoplasty was performed in 5 of 25 (20%) eyes from the povidone-iodine group (median study visit: week 4) and 2 of 24 (8%) eyes from the control group (median study visit: week 2) (*P* = 0.42; Fig. 2). No significant differences in adverse events between the two treatment arms were documented in the adverse event reporting forms established for the trial (Table 2). However, four participants experienced severe ocular discomfort and burning after instillation of 2.5% povidone-iodine, which led to poor adherence with the entire eyedrop regimen. The study doctors and investigators agreed to reduce the concentration to 1% partway through the study in an attempt to minimize poor drug adherence due to intolerance (i.e., 2.5% concentration for the initial 16 participants and 1% concentration for the final 5 participants in the povidone-iodine arm). Non-pre-specified subgroup analyses did not find significantly different results based on whether participants were being randomized to 2.5% or 1% povidone-iodine, nor when stratified by baseline visual acuity or *Acanthamoeba* culture positivity (Table 3).

**TABLE 2:** Adverse event by treatment arm

	Treatment arm		
Adverse event	Control (*n* = 24)	Povidone-iodine (*n* = 25)	*P* value^*a*^
Intraocular pressure > 35 mmHg	1 (4%)	0 (0%)	0.49
Perforation	1 (4%)	1 (4%)	1.00
Therapeutic keratoplasty	2 (8%)	5 (20%)	0.42
Hospitalization	1 (4%)	0 (0%)	0.49

^*a*^ Fisher’s exact test.

**TABLE 3:** Subgroup analyses^*a*^

		Culture-positive from corneal scrapings, week 4		
Subgroup	No.	Control	Povidone-iodine	IRR
Study period				
2.5% povidone-iodine	39	3/12 (25%)	4/13 (31%)	0.98 (0.29–3.26)
1% povidone-iodine	10	2/4 (50%)	2/5 (40%)	0.80 (0.17–3.70)
Baseline visual acuity				
20/400 or better	22	3/7 (43%)	2/10 (20%)	0.41 (0.09–1.84)
Worse than 20/400	27	2/9 (22%)	4/8 (50%)	1.80 (0.46–7.00)
Baseline *Acanthamoeba* culture				
Culture-positive	38	5/12 (42%)	6/14 (43%)	1.03 (0.41–2.57)
Culture-negative	11	0/4 (0%)	0/4 (0%)	NA^*b*^

^*a*^ The incidence rate ratio (IRR) was estimated in a repeated measures analysis using all data from weeks 1, 2, and 4. For brevity, the fraction of each subpopulation with a positive culture is shown only for the week 4 visit; these values do not sum to the total number in the analysis due to the absence of culture data for some participants.

^*b*^ NA, not applicable.

## DISCUSSION

In this study, the addition of adjunctive topical povidone-iodine to the biguanide agent chlorhexidine did not lead to marked differences in any of the pre-specified outcomes over the first month of treatment. The rate of *Acanthamoeba* clearance detected by culture was similar in the povidone-iodine and control groups over the initial 4 weeks of therapy, with approximately two-thirds of eyes in each group clearing *Acanthamoeba* by 4 weeks. The modest sample size precluded a definitive result, with the 95% confidence interval consistent with as much as a 65% reduction in culture positivity in the povidone-iodine group at week 4, or a 129% increase. Clinical characteristics, including time to re-epithelialization and BSCVA, were not significantly different in the two groups, with re-epithelialization observed by 4 weeks in 16% of the povidone-iodine plus chlorhexidine group and 21% of the chlorhexidine-only control group, and 4-week BSCVA of approximately 20/500 in the povidone-iodine group and 20/600 in the control group. Povidone-iodine 2.5% caused pain and burning for some participants, leading to the reduction of the concentration to 1% partway through the trial.

Povidone-iodine is widely used as a pre-operative antiseptic in ophthalmology due to its broad antimicrobial activity and favorable safety profile (18). Povidone is hydrophilic, allowing the transfer of free iodine directly to the target cell membrane, with subsequent oxidation of key pathogen proteins and inactivation of bacterial toxins. The American Academy of Ophthalmology recommends a 5% povidone-iodine solution prior to cataract surgery, although more dilute formulations have higher concentrations of free iodine and have been found to have faster kill times for a variety of organisms (19–21). The effectiveness of povidone-iodine as an antimicrobial for bacterial and fungal corneal ulcers has been assessed in several randomized trials, with a null result in each case. For example, patients with clinical infectious keratitis treated with antibiotics and randomized to adjunctive 2.5% povidone-iodine had similar rates of low vision compared with patients treated with antibiotics alone (22). A single instillation of 5% povidone-iodine in eyes with a corneal ulcer did not result in a reduction of colony-forming units when cultures were taken 10 min later (23). Patients with microbiologically confirmed bacterial keratitis randomized to povidone-iodine 1.25% or neomycin-polymyxin B-gramicidin had no difference in cure rates (24). These studies failed to show a benefit of povidone-iodine beyond antibiotic therapy, perhaps because antibiotics are generally extremely effective at eradicating bacteria from the ocular surface within days of exposure (25, 26). In contrast, months of therapy are often required for *Acanthamoeba* keratitis, highlighting the need for more effective antiamoebic therapies (17, 27).

Several laboratory studies have found povidone-iodine to be a powerful cysticidal agent *in vitro*, with a minimum cysticidal concentration comparable to the biguanide agents that form the basis of AK therapy (7–12). Cysts exposed to povidone-iodine *in vitro* develop morphological changes to the outer wall, destruction of the plasma membrane, and damaged intracellular structures, suggesting potent antiamoebic activity (7, 11). Povidone-iodine was an attractive treatment possibility for AK because it is inexpensive and easily stored, making it suitable for use in many settings. Here, we found that adjunctive povidone-iodine did not hasten *Acanthamoeba* clearance from the corneal surface when used alongside topical chlorhexidine for AK at two eye hospitals in South India. Corneal penetration has been shown to be poor for povidone-iodine, but we hypothesized that any epithelial defect or epitheliopathy present in AK might enhance the entry of povidone-iodine into the stroma (15, 28). The poor penetration of this agent may have limited its clinical efficacy, although the similar rate of culture positivity from corneal scrapings did not provide evidence of a substantial benefit, even in the superficial cornea where penetration would be less of an issue. Nonetheless, the efficacy of povidone-iodine could be different when used in milder infections or infections confined to the epithelial layers, where penetration would presumably be better.

Few trials of antimicrobials for corneal ulcers have implemented a design in which participants underwent repeated corneal scraping to assess the effectiveness of the antimicrobial. A study of bacterial ulcers did repeat corneal scraping 10 min after application of povidone-iodine (23). Repeat corneal scraping was done 6 days after institution of therapy in the Mycotic Ulcer Treatment Trial, which revealed that participants randomized to natamycin were less likely to have fungus identified on culture compared with those treated with voriconazole (29). Trials assessing cross-linking and intrastromal voriconazole injections for fungal keratitis found no difference between treatment groups on repeat cultures done 1, 3, or 5 days after starting treatment, and a trial of rose bengal photodynamic therapy for non-bacterial ulcers showed no difference when culture was repeated at day 2 (30–32). Notably, the microbiological results from these trials were all consistent with the results of clinical outcomes. Follow-up microbiological assessment can be challenging to implement, yet it provides perhaps the most relevant type of evidence when trying to assess the effectiveness of an antimicrobial. Moreover, microbiological cure has been shown to be strongly associated with a host of important clinical outcomes, including visual acuity, scar size, and chance of perforation (33). A key limitation of using corneal scrapings as an outcome, especially for antiamoebic agents with relatively poor corneal penetration, is the possibility that the medications may kill organisms in the superficial cornea but leave viable cysts in the deeper cornea. While this could theoretically cause bias, it would seem to be most problematic if a trial found a marked improvement in *Acanthamoeba* clearance in one of the treatment groups, since it would be unclear if this represented clearance only from the surface or from the entire cornea. But in the case of this trial, adjunctive povidone-iodine did not accelerate *Acanthamoeba* clearance even in the superficial corneal scrapings, suggesting the choice of primary outcome was unlikely to have caused bias for this particular trial. Moreover, repeated cultures have advantages for clinical trial design, providing a relatively objective outcome for which outcome assessors can easily be masked.

This study has limitations. The relatively small sample size, together with missing microbiological data for some participants, limited our ability to detect small differences between the treatment groups. Enrolling a larger sample was difficult due to the paucity of cases seen even at high-volume centers like Aravind, and it was thought that a large effect size might be possible given promising laboratory data (9). The modest sample size increased the likelihood of imbalances in baseline participant characteristics, and while characteristics were generally well balanced, the povidone-iodine group was slightly more likely to have a ring infiltrate at baseline. If the povidone-iodine group started off with more severe ulcers, the observed treatment effect may have been diminished. Study participants and doctors were not masked because the distinct color of povidone-iodine made it difficult to find a suitable placebo eye drop, potentially introducing bias. Adherence to povidone-iodine—assessed only by self-report—was not perfect. Participants reported taking less povidone-iodine than chlorhexidine, even though both medicines were prescribed at the same frequency. While the reported frequency of povidone-iodine was likely adequate (e.g., on average 11 times per day during the first week and 7 times per day during the fourth week), it is possible that a higher dosing frequency could have been more effective. Imperfect adherence in some cases was due to stinging upon instillation, which led us to reduce the concentration of povidone-iodine partway through the study, causing heterogeneity in the intervention. We wanted to use a sufficiently high concentration of povidone-iodine given its poor penetration and settled on 2.5% based on numerous studies that had found this concentration to be safe (16). It is possible that the frequent administration of 2.5% povidone-iodine in this study caused more toxicity than prior studies using less frequent dosing. Study doctors were allowed to start additional antiamoebic medications, and while this could have theoretically introduced bias, there was little evidence of differential antiamoebic use, with 12% of the povidone-iodine group and 8% of the control group starting an additional antiamoebic. Corneal culture and smear are only able to assess AK at the ocular surface, so it is possible that viable cysts were still present deeper in the stroma that could not be detected by these tests. Moreover, these microbiological tests do not have perfect sensitivity and specificity, which may have caused misclassification and reduced the statistical power of the study. Finally, the generalizability of this trial to settings outside these two eye hospitals in South India is not clear, especially since AK observed in India—although caused by similar strains of *Acanthamoeba*—is typically due to agricultural trauma and not contact lens wear and often has a more advanced clinical presentation, frequently with posterior stromal involvement by the time treatment is started (34–36). This study did not specifically assess the role of povidone-iodine when used in the absence of a biguanide agent, when used in earlier infections, or when used in cases localized to the epithelium.

In conclusion, adjunctive povidone-iodine did not lead to a substantial acceleration of *Acanthamoeba* clearance or clinical resolution over the initial month of therapy among individuals with microbiologically confirmed AK being treated with chlorhexidine, although the trial was not powered to detect a smaller yet still clinically meaningful difference. It is possible that different dosing regimens or longer courses of therapy would show a different result. A larger trial would be able to detect a more modest effect, although enrollment would be challenging for this rare infection. Additional therapeutic options would be helpful for the management of AK, given the typically months-long treatment courses needed with currently available antiamoebic agents.

## SUPPLEMENTAL MATERIAL

Supplemental material aac.01512-25-s0001.docx Table S1; Fig. S1.

ASM does not own the copyrights to Supplemental Material that may be linked to, or accessed through, an article. The authors have granted ASM a non-exclusive, world-wide license to publish the Supplemental Material files. Please contact the corresponding author directly for reuse.
