# Supplementary material for: Adjunctive povidone-iodine for Acanthamoeba keratitis: a randomized trial
Source: Antimicrob Agents Chemother. 2026 Mar 31;70(5):e01512-25. doi: 10.1128/aac.01512-25 (PMC13148041; doi:10.1128/aac.01512-25)

**Table of Contents**

[Table S1. Treatment adherence to study medications. 2](#_Toc222214642)

[Figure S1. Predicted probability of a positive acanthamoeba culture. 3](#_Toc222214643)

Table S1. Treatment adherence to study medications. Number of times per day the participant reported taking study medications at each study visit.

|  | Control | | |  | Povidone iodine | | |
| --- | --- | --- | --- | --- | --- | --- | --- |
|  |  | Frequency (times per day) | |  |  | Frequency (times per day) | |
| Visit | No. | Chlorhexidine | Povidone Iodine |  | No. | Chlorhexidine | Povidone Iodine |
| Week 1 | 24 | 12.9 (4.8) | 0 |  | 23 | 13.6 (5.5) | 11.1 (7.9) |
| Week 2 | 24 | 11.0 (4.9) | 0 |  | 22 | 10.7 (5.4) | 9.3 (6.3) |
| Week 4 | 21 | 9.1 (2.7) | 0 |  | 23 | 8.7 (5.3) | 7.1 (6.1) |

Figure S1. Predicted probability of a positive acanthamoeba culture. Predicted probabilities were computed from a repeated measures Poisson regression model at the study time points, with the povidone iodine group shown in orange and the control group in green.


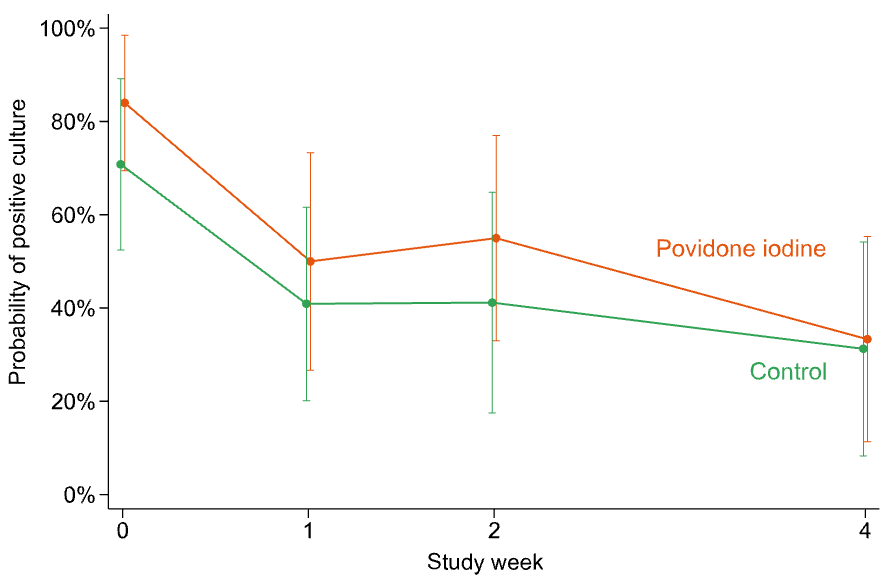

Supplement: Supplemental material — Table S1; Fig. S1. [file aac.01512-25-s0001.docx]
